# Hyperspectral analysis to assess gametocytogenesis stage progression in malaria-infected human erythrocytes

**DOI:** 10.1117/1.JBO.30.2.023516

**Published:** 2025-01-24

**Authors:** Ik Hwan Kwon, Ji Youn Lee, Fuyuki Tokumasu, Sang-Won Lee, Jeeseong Hwang

**Affiliations:** a Korea Research Institute of Standards and Science, Division of Biomedical Metrology, Nanobio Measurement Group, Daejeon, Republic of Korea; b Korea Research Institute of Standards and Science, Division of Biomedical Metrology, Biometrology Group, Daejeon, Republic of Korea; c Chungnam National University, Graduate School of Analytical Science and Technology, Daejeon, Republic of Korea; d Gunma University, Graduate School of Health Sciences, Department of Laboratory Sciences, Maebashi, Japan; e Institute of Tropical Medicine (NEKKEN), Division of Shionogi Global Infectious Diseases Division, Department of Cellular Architecture Studies, Nagasaki, Japan; f Nagasaki University, School of Tropical Medicine and Global Health, Nagasaki, Japan; g University of Science and Technology, Department of Applied Measurement Science, Daejeon, Republic of Korea; h National Institute of Standards and Technology, Applied Physics Division, Boulder, Colorado, United States

**Keywords:** malaria, *Plasmodium falciparum*, erythrocyte, gametocyte, gametogenesis, hyperspectral imaging, spectral angle mapper, hemoglobin, hemozoin

## Abstract

**Significance:**

Developments of anti-gametocyte drugs have been delayed due to insufficient understanding of gametocyte biology. We report a systematic workflow of data processing algorithms to quantify changes in the absorption spectrum and cell morphology of single malaria-infected erythrocytes. These changes may serve as biomarkers instrumental for the future development of antimalarial strategies, especially for anti-gametocyte drug design and testing. Image-based biomarkers may also be useful for nondestructive, label-free malaria detection and drug efficacy evaluation in resource-limited communities.

**Aim:**

We extend the application of hyperspectral microscopy to provide detailed insights into gametocyte stage progression through the quantitative analysis of absorbance spectra and cell morphology in malaria-infected erythrocytes.

**Approach:**

Malaria-infected erythrocytes at asexual and different gametocytogenesis stages were imaged through hyperspectral confocal microscopy. The preprocessing of the hyperspectral data cubes to transform them to color images and spectral angle mapper (SAM) analysis were first used to segment hemoglobin (Hb)- and hemozoin (Hz)-abundant areas within the host erythrocytes. Correlations between changes in cell morphology and increasing Hz-abundant areas of the infected erythrocytes were then examined to test their potential as optical biomarkers to determine the progression of infection, involving transitions from asexual to various gametocytogenesis stages.

**Results:**

Following successful segmentation of Hb- and Hz-abundant areas in malaria-infected erythrocytes through SAM analysis, a modest correlation between the segmented Hz-abundant area and cell shape changes over time was observed. A significant increase in both the areal fraction of Hz and the ellipticity of the cell confirms that the Hz fraction change correlates with the progression of gametocytogenesis.

**Conclusions:**

Our workflow enables the quantification of changes in host cell morphology and the relative contents of Hb and Hz at various parasite growth stages. The quantified results exhibit a trend that both the segmented areal fraction of intracellular Hz and the ellipticity of the host cell increase as gametocytogenesis progresses, suggesting that these two metrics may serve as useful biomarkers to determine the stage of gametocytogenesis.

## 1 Introduction

Malaria is a life-threatening infectious disease caused by parasites spread by bites from infected *Anopheles* mosquitoes. Global warming speeds up the growth of parasites in the mosquito, resulting in not only accelerated transmission in tropical regions but also the reemergence of cases in regions that were formerly malaria-free.^1^ The slowdown of malaria control efficiency, the large number (more than 600,000) of annual deaths, and an increase in drug-resistant parasites^2^ demand more compliant drug development strategies, which are pivotal in combating this deadly disease at every stage of the infection. The development of antimalarial drugs has primarily focused on asexual blood-stage parasites as parasites in this stage directly cause symptoms in patients. For a more comprehensive antimalarial strategy, drug developers have recently paid more attention to stage-dependent malaria studies focused on the maturation process of gametocytes, or gametocytogenesis,^3^^–^^5^ which has opened other drug design paradigms toward the development of anti-gametocyte drugs. The biology of gametocytes differs from that of asexual-stage parasites in many ways, manifested by progressive changes in biochemical, morphological, and mechanical properties during gametocytogenesis.

Morphology and changes in the intracellular molecular species involved in the metabolic transitions of parasites are important metrics to assess the progression of gametocytogenesis.^6^ Accurate determination of the stage of gametocytogenesis with a nondestructive optical method enables the assessment of the efficacy of anti-gametocyte drugs by measuring property changes that confirm the regulation of parasite growth without further stage progression. Within host erythrocytes, the main source of amino acids for blood-stage *Plasmodium* growth is hemoglobin (Hb), where the parasite’s metabolic byproduct is hemozoin (Hz).^7^^,^^8^ The difference in the absorption spectra between Hb and Hz allows for optical measurements of the metabolic transitions involved in infectious stage progression by spectroscopy or spectroscopic imaging. Quantitative spectroscopic measurements of such transitions require an accurate assessment of the absolute quantity of each molecular species. For this, exogenous labeling is not desirable as it would obscure the optical properties of the metabolites, posing a challenge to quantitative evaluation.

Various label-free imaging technologies have been utilized for the detection and study of malaria-infected erythrocytes. Examples include infrared imaging spectroscopy to measure distinctive lipid signatures associated with the different stages of malaria parasites;^9^ attenuated total reflectance infrared spectroscopy to detect and quantify early-stage malaria parasites in infected erythrocytes;^10^ quantitative phase imaging to detect schizont stage–infected cells without staining;^11^ multispectral-multimodal optical analysis for malaria diagnostics;^12^ and multispectral nanoimaging of malaria-infected erythrocytes.^13^ In addition, hyperspectral imaging has been demonstrated for various biological and biomedical applications,^14^^,^^15^ especially for the detection of Hb and Hz in malaria-infected erythrocytes.^16^

Here, we report an application of hyperspectral confocal microscopy of *Plasmodium falciparum* (*Pf*)-infected erythrocytes to delineate the gametocytogenesis stage-dependent optical properties of the infected cells. Measurements of the intracellular spatio-spectral changes because of progressive Hb consumption and Hz production were conducted to test their potential as stage-defining biomarkers of gametocytogenesis. In addition, as gametocytes at later stages are identified by their banana-shape appearance concomitantly with elongated host erythrocytes,^17^ we also quantified the morphological changes of the host erythrocytes and evaluated the correlation between cell morphology and Hz content at various stages. The primary focus of this study is to develop and validate an image analysis pipeline toward its application for the quantitative study of the biology of malaria infection in human erythrocytes. For these quantitative analyses, we harnessed a comprehensive workflow with image processing algorithms to analyze the stage-dependent spectral and morphological properties from hyperspectral data cubes of single parasitized human erythrocytes.

## 2 Methods

### 2.1 Preparation of Malaria-Infected Erythrocytes for Imaging

*In vitro* asexual-stage culture of 3D7 line *Pf* was conducted in Roswell Park Memorial Institute (RPMI) 1640 media supplemented with 0.5% Albumax II (Invitrogen, Waltham, Massachusetts, United States), 2-mg/mL sodium bicarbonate (Invitrogen), 0.10-mM hypoxanthine (Sigma–Aldrich, St Louis, Missouri, United States), 25-mM HEPES, and 10-mg/mL gentamicin (Gibco, Carlsbad, California, United States) at 37°C, at 5% hematocrit (Hct). Differentiation into gametocytes was induced by setting up separate cultures at 0.1% parasitemia with 6% Hct supported by 10% human serum instead of Albumax II. On day 3, Hct was reduced to 3%, and the remaining asexual parasites were eliminated by 3-day treatment (days 9 to 11) with 50-mM N-acetylglucosamine (NAG). Glass coverslips were cleaned with a mixture of sulfuric acid and hydrogen peroxide (4:1 volume ratio) and rinsed with distilled water (DW). For attachment of cells, the coverslips were coated with diluted poly-l-lysine for 10 to 15 min, washed with DW, and dried. Cell suspension in RPMI 1640 without serum was applied to the coverslips, incubated for 10 to 30 min, and then rinsed with the RPMI without serum. Cells were fixed with 2% and 4% paraformaldehyde (PFA) sequentially, followed by quenching unreacted PFA with 0.1-M glycine in 1× phosphate buffered saline (PBS). The coverslips were then placed face down on glass slides and sealed with vacuum grease around the edges to prevent PBS from leaking.

### 2.2 Hyperspectral Confocal Microscopy

Hyperspectral data cubes were acquired with a confocal laser scanning microscope (TCS SP5 II, Leica Microsystems, Mannheim, Germany) equipped with a supercontinuum laser source (NKT Photonics, Birkerod, Denmark). Images were obtained with an achromatic oil immersion lens (HCX PL APO 100X 1.44 NA) and with a photomultiplier (PMT) detector (Air-cooled R9624 Hamamatsu Photonics, Bridgewater, New Jersey, United States) in transmission mode. Data cubes of the regions of interest (ROIs) with uninfected and/or *Pf*-infected erythrocytes were acquired in illumination wavelength scan mode with 61 bands, from 400 to 700 nm with a step size of 5 nm. The wavelength scan was performed using an acousto-optical beam splitter tunable filter in the illumination beam path. The ROI size was controlled by the scan range of the X−Y piezo stage of the confocal microscope.

### 2.3 Data Cube Analysis

Our data cube analysis algorithm adopted the following workflow of image processing techniques: (1) conversion of hyperspectral data cubes to standard red, green, and blue (sRGB) images for initial human visualization of the multiband data cubes to tabulate infected and uninfected erythrocytes in a group of cells, which allows for selecting the infected cells for further hyperspectral analyses; (2) elimination of achromatic out-of-focus image segments by an extended depth of field (EDOF) algorithm to identify cell boundaries for morphological analysis; and (3) spectral angle mapper (SAM)-based hyperspectral analysis and morphological analysis of the infected erythrocytes to quantify their stage-dependent chemical composition and morphological changes, respectively. Our algorithms were developed in Python (Anaconda, Austin, Texas, United States), and the results were verified with corresponding MATLAB (Mathworks, Natick, Massachusetts, United States) built-in functions for the key equations below.

### 2.4 Converting Hyperspectral Data Cubes to sRGB Images

Hyperspectral data cubes were converted to sRGB images by a color space model introduced by the International Commission on Illumination 1931 (Commission Internationale de l’Eclairage 1931, CIE 1931), which was later standardized by the International Electrotechnical Commission.^18^ The conversion ensures the closest match of the multiband spectrum to a human-perceived color. In brief, for a given spectrum at pixel position (n,m) of data cube I(n,m,λ), its conversion to sRGB(n,m) is given by

$$\mathrm{sRGB}(n,m)=\gamma M\cdot (\underset{\lambda }{\sum }I(n,m,\lambda )\cdot [\begin{array}{c} X_{\lambda^{\prime }}\\ Y_{\lambda^{\prime }}\\ Z_{\lambda^{\prime }} \end{array}]),$$ (1)

where the elements of the column matrix $\left[\begin{array}{c} X_{\lambda^{\prime }}\\ Y_{\lambda^{\prime }}\\ Z_{\lambda^{\prime }} \end{array}\right]$ are CIE XYZ values at the standard wavelength, $\lambda^{\prime }$ is the closest match to λ, and M and γ   are the transformation matrix from CIE XYZ to sRGB color space and gamma correction, respectively, which are defined elsewhere.^19^

### 2.5 Extended Depth of Field

The EDOF technique was introduced to significantly broaden the spatial depth of field in imaging an objective. Adaptive optics have been used to achieve EDOF at the device level,^20^ but as a complementary technique, a variety of image processing algorithms have also been developed to achieve the same goal.^21^ The EDOF algorithm in essence reconstructs the single best-focused image from a group of N images, $\left\{I_{n};n=1\ldots N\right\}$, obtained from the same field of view where some images in the group are out of focus. The best-focused image, F(n,m), is given by

$$F(n,m)=I({\arg\;\max}_{\left(n,m\right)}F(I_{n}(n,m))),$$ (2)

where F is an image processing filter to enhance or maximize the intensity associated with the target features of interest. In broadband hyperspectral microscopy, some spatial features often become out of focus in a certain wavelength range due to the chromatic aberration of the microscope. In this study, resolving the boundary of the cells is necessary to measure their shapes for morphological analysis, and therefore, we chose the Laplacian filter, a second-order derivative operator known as an effective spatial filter for edge detection by enhancing the intensity gradient at a high spatial frequency.

### 2.6 Spectral Angle Mapper

The SAM algorithm computes the spectral angle (SA) between a sample spectrum $\vec{I}(m,n)$ and $\vec{R^{\left(k\right)}}$, the reference spectrum of the given molecular species k. The SA at pixel (m,n) with respect to $\vec{R^{\left(k\right)}}$ is given by

$$\alpha_{k}(m,n)=\cos^{-1}(\frac{\overset{\to }{I}(m,n)\cdot \overset{\to }{R^{\left(k\right)}}}{\left\|\overset{\to }{I}(m,n)\right\|\times \|\overset{\to }{R^{\left(k\right)}}\|})=\cos^{-1}(\frac{\sum_{i=1}^{N}I_{i}(m,n)R_{i}^{\left(k\right)}(m,n)}{\sqrt{\sum_{i=1}^{N}I_{i}(m,n{)}^{2}}\sqrt{\sum_{i=1}^{N}R_{i}^{\left(k\right)}(m,n{)}^{2}}}),$$ (3)

where $I_{i}$ and $R_{i}$ are the spectral values in the i’th band (wavelength) of the sample and reference spectrum, respectively. This SAM technique allows the generation of a two-dimensional (2D) intensity image or segmented map with respect to a specific reference spectrum, where the intensity is scaled with the SA or the spectral similarity to the reference spectrum. The SA quantifies the similarity of $\vec{I}(m,n)$ to $\vec{R^{\left(k\right)}}$: the lower the α value, the higher the similarity.

## 3 Results and Discussion

### 3.1 Preprocessing of Hyperspectral Images and Extraction of Reference Spectra

**Fig. 1:**
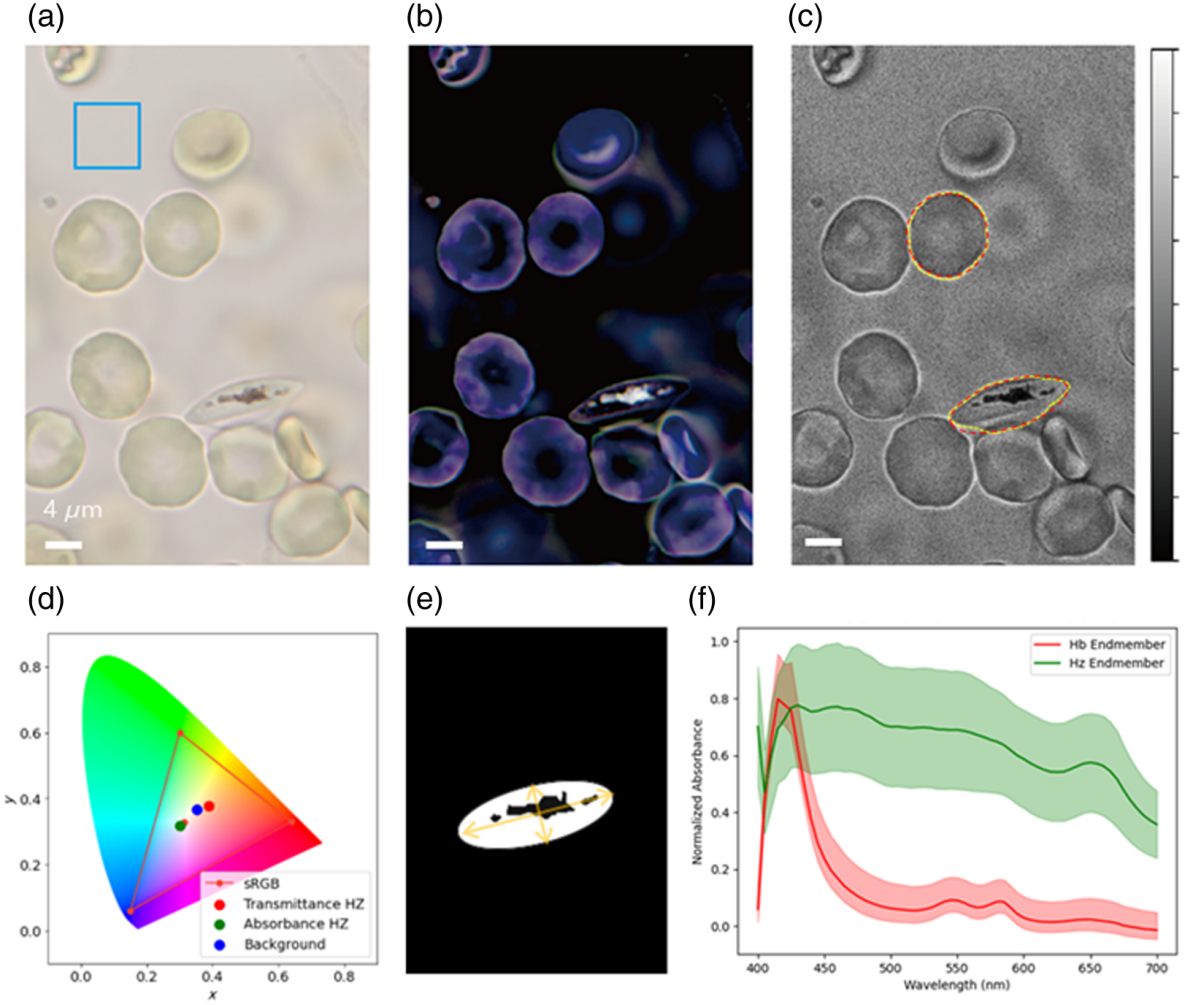
Preprocessing of hyperspectral images and extraction of hemoglobin and hemozoin reference spectra. (a) sRGB image converted from a hyperspectral data cube. (b) Absorbance sRGB image generated by subtracting the background marked with the blue box in panel (a). (c) EDOF image used for masking the cell ROIs. The yellow and red lines mark the cells and fitted ellipsoids, respectively. (d) sRGB color space in the CIE 1931 chromaticity x−y diagram. The red and green dots show the color difference in the Hz-abundant area between the transmittance and absorbance sRGB images. The blue dot represents the background color of the sRGB image shown in panel (a). (e) Fitted ellipsoid and intracellular Hz-abundant regions of an infected cell. (f) Extracted reference spectra for Hb (red) and Hz (green) with a standard deviation fitting uncertainties shown as shaded regions.

Figure 1(a) shows an sRGB image transformed from a data cube of a group of erythrocytes including a *Pf*-infected erythrocyte. This transformation, from a hyperspectral data cube to a human-perceptible sRGB color image, enables direct identification of infected erythrocytes among other cells by their intracellular dark spots, attributed to the strong light absorbance of Hz associated with the intracellular parasites. Conversion to sRBG images allows for visual identification of infected cells to ensure classification between infected and uninfected cells for further hyperspectral analyses of different cell types. To quantify the intracellular Hz content, an absorbance data cube, A(m,n), was obtained from the hyperspectral data cube by

$$\overset{\to }{A}=A(m,n)=-\log(\frac{\overset{\to }{I}-I_{\text{dark}}}{I_{o}-I_{\text{dark}}}),$$ (4)

where $I_{o}$ is the spectrum of the incident light, an averaged spectrum from a selected region with no cells [blue square in Fig. 1(a)], and $I_{\text{dark}}$ is the dark background or weak ambient signal with the illumination source off. The processed sRGB image of the data cube $\vec{A}$ shown in Fig. 1(b) further enables the visualization of the Hz-abundant intracellular region characterized by high absorbance via the bright pixels, which correspond to the dark spots in the sRGB image in Fig. 1(a). This correlation justifies the selection of a *Pf*-infected cell based on the presence of intracellular dark spots in the sRGB image. Moreover, the sRGB color coordinates of the transmittance and absorbance of Hz-abundant areas can be represented in a chromatic diagram from CIE XYZ values, as shown in Fig. 1(d). In this diagram, the x and y coordinates represent the chromaticity coordinates, which denote the hue and saturation of the colors independent of the luminance signal. The transmittance Hz coordinates differ from the background illumination by 10.49% in the x coordinate and 3.20% in the y coordinate, indicating a strong influence of the background light. However, the absorbance Hz coordinates, with the background removed, more clearly reveal the intrinsic Hz spectrum by its color appearance. Therefore, analyzing Hz-abundant areas using the intrinsic absorbance spectrum allows for clearer identification than with the transmittance spectrum.

**Fig. 2:**
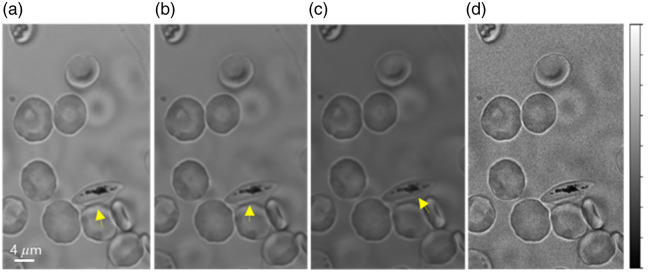
EDOF images created from hyperspectral data cubes. (a)–(c) Spectral images of cells at 420, 520, and 620 nm, respectively. Yellow arrows present the focusing difference at cell edges. (d) Generated EDOF image showing a well-defined cell edge.

The other key goal of this work is to correlate the morphological changes of cells with the progressive infection stage as the parasite grows within the host cell. We found that the cell’s morphology changes from a torus-like shape to an elongated form as the infection stage progresses. To quantify this stage-dependent change, the cell boundaries at various infection stages were fitted to an ellipsoid, from which the ellipticity was calculated by dividing the length of the minor axis of the fitted ellipsoid by the major axis. An initial attempt to define the continuous cell boundary from the local maxima around the cell from a single image at one wavelength was not successful as chromatic aberration blurs the segments of the cell boundary in a single image [see the arrows in Figs. 2(a)–2(c)] as some parts of the cell membrane in 3D cell become out of focus at certain wavelengths. To address this issue, the EDOF technique allowed us to build a single focus-enhanced image exhibiting a continuous cell boundary, namely, a bright line around its edge, as in Fig. 2(d). We then applied the Hough spatial filter to this enhanced image to find the pixels with a local maximum along the boundary [yellow lines in Fig. 1(c)], followed by fitting to an ellipsoid [red dashed lines in Fig. 1(c)] and calculating the ellipticity of the cell shape. This ellipsoid was also used to calculate the total projected or “masked” cell area as well. Similarly, EDOF processing also allowed the definition of the boundary of the segmented Hz-abundant regions, determined by the threshold SA (TSA) with respect to the Hz reference spectrum. Figure 1(e) displays the fitted ellipsoid region and intracellular Hz-abundant region. A spectrum of Hz molecules is shown in Fig. 1(f), which was averaged from Hz-rich regions in 40 different parasite-infected erythrocytes. For the Hb spectrum shown in Fig. 1(f), we averaged the spectra from 10 uninfected cells over their entire intracellular area. These averaged spectra of Hb and Hz were used as reference spectra to segment the intracellular regions with abundant Hb and Hz, respectively. Details of this segmentation procedure are discussed in Sec. 3.2.

### 3.2 Segmentation of Intracellular Hb and Hz Distributions by the SAM Technique

Hemozoin, a biocrystal synthesized by parasites for detoxification, is the key molecule to understanding the life cycle of parasites as well as identifying the parasite subspecies and diagnosing and targeting antimalarial compounds.^5^ Among other characteristics, its size and distribution inside a parasitized erythrocyte vary for different *Plasmodium* species, making these properties important biomarkers for antimalarial drug development and testing.^2^^,^^22^ Hz-abundant regions are larger in *Pf* gametocytes than in the asexual stage. Therefore, changes in the amount of intracellular Hz provide an important biomarker for the staging of malaria infection. In erythrocytes cultured with *Pf*, we observed a decrease in Hb abundance with a concomitant increase in Hz as the parasites grew and replicated inside the cell. Quantification of this trend was possible with the SAM results of Hb and Hz at various infection stages. In essence, the SA computed with respect to the reference spectra of Hz and Hb in Fig. 1(e) can be used to segment the regions with abundant Hb or Hz from the hyperspectral data cube.

**Fig. 3:**
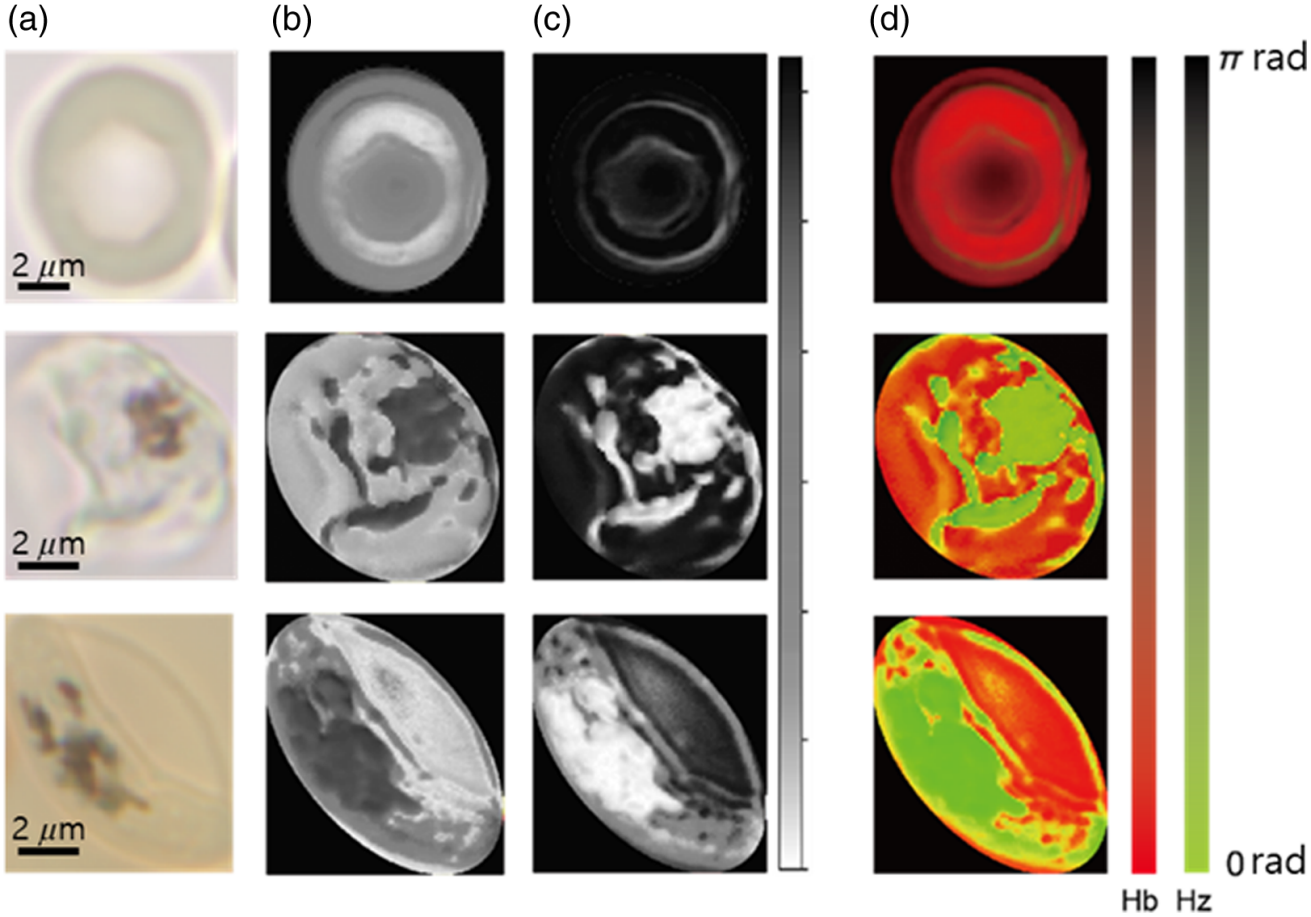
Comprehensive analysis of hemoglobin and hemozoin overlap via SAM analysis. The first row shows uninfected cells, whereas the second and third rows present the spectral angle maps of infected cells at the asexual stage and stage V, respectively. (a) Converted sRGB images from hyperspectral data cubes. (b) SAM results using the reference spectra of Hb from Fig. 1(e), highlighting regions with spectral signatures such as Hb. (c) SAM results using the reference spectra of Hz from Fig. 1(e), highlighting regions with spectral signatures like Hz. (d) Overlaid SAM results as RGB images. Red and green channels represent the SAM results shown in panels (b) and (c), respectively.

Figures 3(b) and 3(c) are SA maps segmented for Hb and Hz, respectively, where the grayscale corresponds to the degree of spectral similarity to each reference spectrum, from 0 rad (white) to π rad (black). Comparing the two grayscale SA maps of an uninfected cell shown in the first row [top of Figs. 3(b) and 3(c)], the SAs with respect to the Hb reference spectrum are significantly lower (brighter in the grayscale) than those of Hz, confirming the dominance of Hb for the entire cell area. In the grayscale SA maps of infected cells shown in the second and third rows, the parasite regions show opposite contrast between Hb and Hz. The bright Hz-abundant regions are scattered across the entire cell at an early stage of infection [middle of Figs. 3(b) and 3(c)] but assemble into a compartmented region at a late stage [bottom of Figs. 3(b) and 3(c)]. In this later stage, Hb is confined within the other intracellular compartment.

Note that the SA maps with respect to Hz in the uninfected cell include regions with substantially small SA values, indicative of non-negligible spectral mixing with the Hz spectrum, although the cell is known not to contain Hz molecules. Hyperspectral confocal microscopy is sensitive to optical scattering from local cellular structures, resulting in the inclusion of complex broadband scattering in the spectra, which requires a strict segmentation condition with a high TSA value for complete exclusion.^16^ Further separation of the scattering contribution from the abundant Hb and Hz would require a model based on their complete physical and optical properties, which is beyond the scope of this work. The spectrum-mixed regions are shown in the two-color overlay images in Fig. 3(d), where the SAs with respect to Hb and Hz are separated into red and green channels, respectively. In the uninfected cell, the SAs of Hz are high, appearing as dark green in the top panel. In the infected cells, the spectrum-mixed (yellow) regions are prominent at the cell boundary and at the interface between Hb- and Hz-abundant regions.

**Fig. 4:**
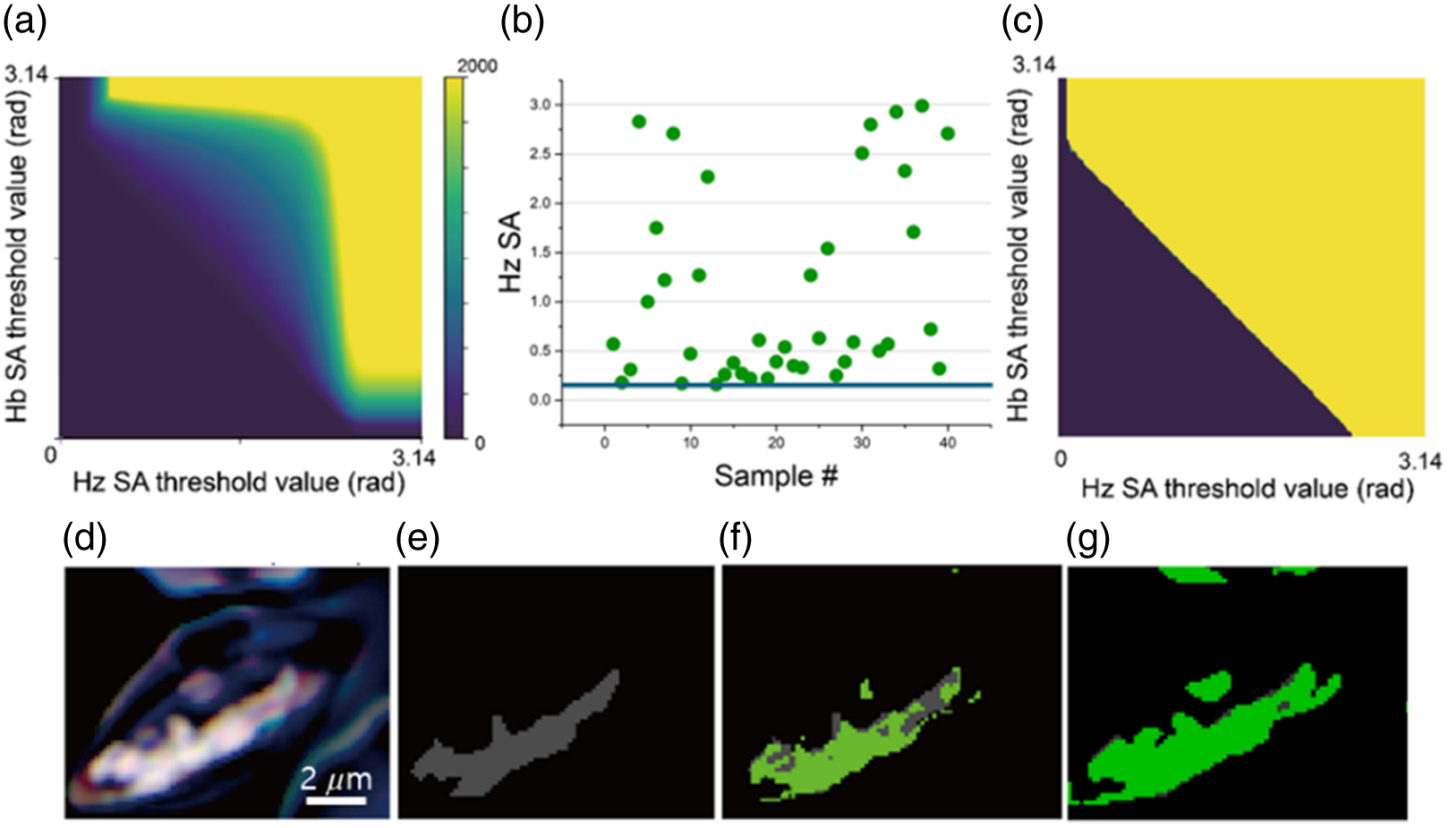
Binary segmentation algorithm to compute the areas exclusively with abundant hemoglobin or hemozoin. (a) Overlap pixel map of Hb and Hz showing threshold spectral angles (TSAs) of SAM results for Hb (horizontal axis) and Hz (vertical axis) with a color gradient indicating intensity levels. (b) Hz TSAs across various samples, visualizing the variability of Hz distribution in each sample to contain the Hz areas. (c) Binary overlap pixel map showing the specific overlap between Hb and Hz at increasing TSA values. (d) Absorbance sRGB image of an infected cell. (e)–(g) Detailed spectral analysis images of Hz at various TSAs demonstrating the frequency of specific angle ranges in spectral matches, guiding the containment of Hz areas. The Hz area appears green under a TSA of 0.01 rad (e), 0.1 rad (f), and 0.26 rad (g).

To quantify the progressive change in abundance from Hb to Hz, a binary segmentation algorithm was applied to compute the areas exclusively with abundant Hb or Hz after eliminating the spectrum-mixed regions. The key to this binary segmentation algorithm is to determine the optimal TSA (OTSA) for Hb and Hz, below which the spectrum-mixed regions are eliminated. To this end, we stepwise increased the TSA for Hb and Hz from 0 rad to π rad by 0.01 rad and computed the number of spectrum-mixed pixels versus TSA, where the pixels are segmented into both Hb and Hz. The TSA-dependent spectrum-mixed pixel numbers from the data cube of the late-stage infected cell in Fig. 1 are displayed in a 2D pixel count map in Fig. 4(a). This map shows that the count of spectrum-mixed pixels decreases as the TSAs decrease, becoming zero when the TSAs are reduced below 0.31 and 0.07 for Hz and Hb, respectively. The largest TSA values below which the counts are 0 define the OTSA values. The TSA value to exclusively include Hz-abundant areas varied considerably among data cubes, ranging from 0.26 to 3.07 rad, as shown in Fig. 4(b). To obtain global OTSA values applicable to all 40 data cubes under consideration, the OTSA values for all data cubes were determined from the interfacial line between the two areas in the plot in Fig. 4(c), where the yellow region corresponds to nonzero counts and the dark blue to zero. The OTSA value with respect to one reference spectrum at a given TSA can be determined from this binary plot. For our exclusive segmentation of Hz for all 40 data cubes, we chose the most stringent OTSA value of 0.26 for Hz and 1.09 for Hb from the plot in Fig. 4(b), ensuring that the spectrum-mixed pixel numbers below these OTSA values are zeros. To validate that the OTSA for Hz effectively segments the parasite region, Figs. 4(e)–4(g) compare binary Hz-segmented maps at various TSA values, where the green segmented region at OTSA = 0.26 coincides well with the gray region with an absorbance of ≥0.5 between wavelengths of 450 to 600 nm.

### 3.3 Cell Morphology Versus Hemozoin Abundance by Gametocytogenesis Stage

Previous studies have discovered that small fractions of asexual parasites switch to gametocytes, the sexual stage of malaria parasites, and undergo a maturation process known as gametocytogenesis over 10 to 15 days. The male and female gametocytes, once picked up by a mosquito, are differentiated into oocysts in their midgut that further develop into sporozoites before being transmitted to another mammalian host. The biology of a gametocyte differs from that of asexual-stage parasites in biochemical and morphological aspects. In particular, gametocytogenesis includes a five-stage morphological change in the gametocyte and its host erythrocyte as well, with a trend of a shape transition from circular to elliptical and eventually a banana-like curvature.^6^ This stage-dependent morphological change was first reported by Hayward et al.^17^ and further studied for microscopic details.^23^ Both the morphology of the host cell and the intracellular distribution of blood-staining Giemsa pigment have been suggested as important biomarkers to identify the species of parasite and gender of gametocyte. For instance, at stage V, the male *Pf* gametocyte appears thicker and the cytoplasm appears pale blue after Giemsa staining, whereas the female is more elongated and curved with a darker blue stained cytoplasm. Furthermore, Giemsa pigment tends to be scattered in male gametocytes while densely coagulated in female gametocytes. In addition, unlike elongated *Pf* gametocytes at later stages, the *Plasmodium vivax* gametocytes are of round shape in both males and females at later stages.

**Fig. 5:**
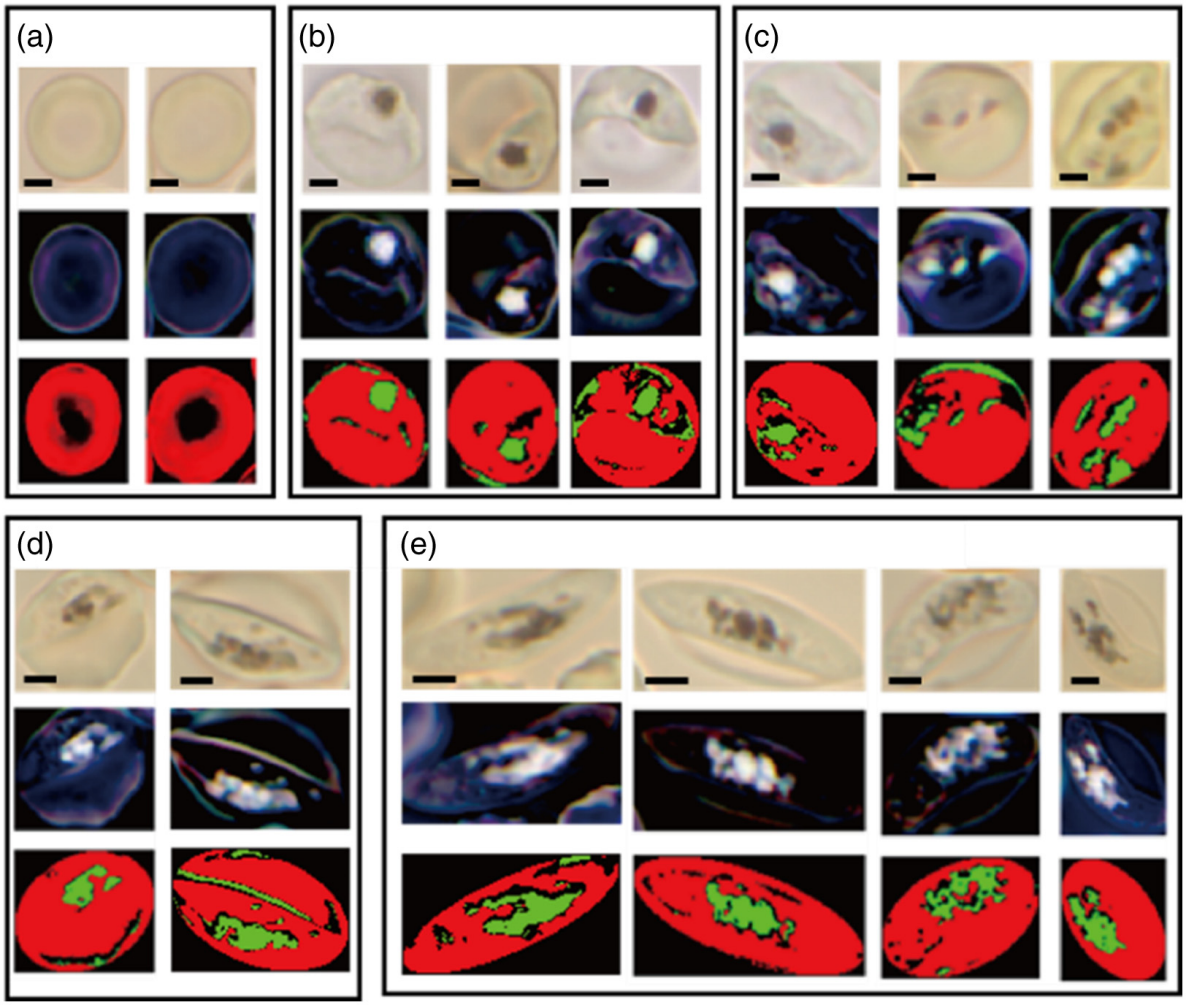
Segmented maps of hemoglobin and hemozoin in erythrocytes at different stages, obtained through the SAM analysis. sRGB images from the hyperspectral dataset, absorbance sRGB images, and binary segmented maps determined by the optimal threshold spectral angle (1.09 for Hb in red and 0.26 for Hz in green) are presented in the top, middle, and bottom row, respectively. (a) Uninfected cells, (b) asexual stage, (c) gametocyte stages 1 and 2, (d) gametocyte stage 3, and (e) gametocyte stages 4 and 5. The scale bars are 2  μm.

Although the Giemsa-stained blood smear test has been the gold standard for malaria detection assays using microscopy, the technique is qualitative as the sensitivity depends on the quality of the imaging device, staining procedures and reagents, and experience of examiners. In this study, we investigated the correlation between the amount of intracellular Hz, instead of Giemsa pigment, and the host cell’s morphology to test if the intracellular abundance of Hz can be used as a biomarker to determine the stage of *Pf* gametocytogenesis. The stage-dependent morphology was quantified by analyzing a total of 43 cells, comprising 21 asexual and 22 gametocytes at various stages, by computing the ellipticity of the cells as discussed above. The Hz abundance was measured by computing the percentage of the number of Hz-abundant pixels against the total number of pixels in the cell. The Hz-abundant coverage was measured after eliminating both Hb-dominant and spectrum-mixed pixels, following the procedures described above. All 43 cells are displayed in Fig. S1 of the Supplementary Material, including sRGB and absorbance images as well as abundance maps of both Hb and Hz. Figure 5 presents the results from selected cells categorized into five groups: uninfected cells (a), asexual stage (b), gametocytes in stages 1 and 2 (c), stage 3 (d), and stages 4 and 5 (e), following the criteria suggested by Day et al.^17^ The difference between the asexual stage and gametocyte stages 1 and 2 is subtle in terms of cell morphology with the same round shape, but gametocyte stages 1 and 2 in the sRGB images in the top row show multiple dark Hz-rich spots segregated into a compartmentalized area, whereas the asexual stage shows only a single dark spot. Note that asexual parasites on days 9 to 11 were treated with N-acetylglucosamine (NAG), so no asexual parasites were present in the culture showing gametocytes. As maturation progresses to gametocyte stage 3, the shape of the Hz-abundant region transforms from round to elongated by the associated scattered dark spots. In stages 4 and 5, the cell starts to become crescent-shaped, and the dark spots spread more broadly, with an increased occupied region. The absorbance images displayed in the middle row exhibit bright spots corresponding to the dark spots in the sRGB images, confirming that the dark spots are light-absorbing intracellular Hz molecules associated with the parasite. This is also confirmed by the red (Hb) and green (Hz) regions in the segmentation results in the bottom row, showing the same distribution pattern.

**Fig. 6:**
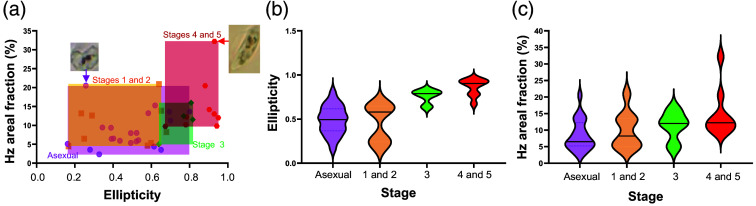
Stage-dependent changes in cell morphology and hemozoin areal fraction. (a) Correlation graph between ellipticity and Hz areal fraction. (b) Ellipticity and (c) Hz areal fraction plots of different stages of infected cells showing stage progression. In panels (b) and (c), the violin plots depict the distribution of ellipticity and Hz areal fraction, respectively, across various stages, whereas the surrounding density plot visualizes the data distribution and density. The horizontal bars indicate the median value.

Overall, the stage-dependent segmented maps in Fig. 5 show a trend that the Hz-segmented areal fraction and ellipticity increase as gametocytogenesis progresses. This is in qualitative agreement with the plot in Fig. 6(a) showing that the rectangular area defined by the range of the Hz-segmented areal coverage and the cell’s ellipticity (yellow for stages 1 and 2, green for 3, and red for 4 and 5) shifts to the upper right as the stage progresses. The data for the asexual stage cover a broad range both in areal fraction and ellipticity, where the areal fraction is below 15% for all cells excluding an outlier with a relatively high areal fraction (purple arrow). The image of the cell corresponding to this outlier data point shows an extended Hz-segmented region indicative of a signature of switching from the asexual to gametocyte stage. The broad distribution in ellipticity is due to various deformed cell shapes. In the stages 4 and 5 group data, an outlier (red arrow) is also identified with a relatively higher areal fraction. This data point corresponds to a cell with more scattered Hz-abundant regions, implying that this cell may be a rare male cell; a female-biased sex ratio is usually observed in gametocytogenesis stages due to the production of a higher percentage of committed female schizonts than males.^24^ Further details of the stage-dependent ellipticity and Hz-segmented areal fraction are shown in Figs. 6(b) and 6(c), exhibiting that ellipticity shows stronger correlation. However, we need more data points for a more meaningful statistical analysis to quantify the trend with higher accuracy. As only a small fraction of asexual-stage parasites committed to switching to gametocytes, further studies are needed with larger-scale incubation, with the anticipation that hyperspectral imaging can provide a more accurate determination of the stage dependency of the biomarkers. In Sec. 4, we outline additional areas for further exploration to utilize hyperspectral imaging for malaria diagnosis and research, obtained through our data acquisition and analysis workflow.

## 4 Conclusions and Perspectives

We have harnessed a workflow of image processing algorithms to analyze hyperspectral data cubes of parasitized human erythrocytes. The workflow enabled the quantification of changes in the relative contents of Hb and Hz in the host cells and cell morphology at various parasite growth stages. The optical properties were quantified by the areal fractions of Hb and Hz in segmentation maps and morphology by the cell ellipticity. The quantified results exhibited a trend that both the segmented areal fraction of intracellular Hz and the ellipticity of the host cell increase as gametocytogenesis progresses, suggesting that these two metrics may serve as useful biomarkers to determine the stage of gametocytogenesis. The trend of ellipticity versus stage is much more pronounced than that of the Hz areal fraction versus stage; this may be due to the increased aggregation of Hz molecules after stage 3. More detailed analysis of local Hz concentrations requires further separation of scattered light signals, which are substantial in microscopy setups. A straightforward way to address this is by rigorous modeling of local scatterers, i.e., Hz crystals, which is challenging without details of the optical and physical properties of Hz crystals in the cell. More advanced segmentation methods such as K-means clustering or automated extraction techniques may provide solutions. K-means clustering automatically groups pixels into clusters based on spectral similarity, reducing the need for manual selection and efficiently identifying distinct spectral features or regions.^25^^,^^26^ This would enable accurate, statistics-based extraction of reference spectra to detect subtle spectral variations in complex light scattering effects. Another approach is to apply machine learning–based automated reference spectra extraction techniques leveraging training datasets with a library of known optical properties of Hz crystals to enable sub-pixel separation of the target signatures.

The absolute concentration change measurement is challenging. The pixel intensity maps in Figs. 3 and 5 cannot be directly related to the stage-dependent Hb consumption quantity without further information on the relation between pixel intensity versus Hb concentration. For now, for qualitative demonstration, Fig. S2(a) in the Supplementary Material exhibits that the mean value of the Hb-segmented area decreases from the asexual to the gametocyte stage and to the later gametocytogenesis stages. However, the broadly distributed data due to the limited number of cells make it challenging to conclude a trend with a statistically meaningful rigor. We have also calculated the averaged pixel intensity change over the cellular stage progression with cell images at 540-nm wavelength, where both Hb and Hz maps show the highest contrast, for the intracellular Hb-segmented areas. Figure 2S(b) in the Supplementary Material exhibits that the mean pixel intensity decreases from the asexual to the gametocyte stage and to the later gametocytogenesis stages except at the stages IV and V, qualitatively suggesting a trend of increasing Hb consumption as the gametogenesis progresses. The rebound of the pixel intensity at stages IV and V may be due to the local thickness increase of the cells, which would increase the intensity in the Hb-segmented map. An accurate assessment needs further investigation with the cell shape information in 3D.

Optical biomarkers to accurately determine the stages of gametocytogenesis are critical in developing anti-gametocyte drugs, which are alternatives to common antimalarial compounds that are not effective at killing sexual-stage parasites (gametocytes). Antimalarial drugs used against gametocytes can effectively block parasite transmission from a human host to a mosquito. Accordingly, accurate determination of the stage of a gametocyte with the nondestructive optical method reported here would enable the assessment of the efficacy of anti-gametocyte drugs as biomarkers can determine if the parasite growth is regulated without further stage progression. In addition, accurate quantification of optical biomarkers may be used as a metric to evaluate the bioactivity of gametocytes. As the main source of amino acids for parasite growth is Hb, long-term stage development of gametocytes compared with asexual stages (2 weeks versus 48 h) should be sufficient to consume most Hb in host erythrocytes. In fact, our previous study of gametocyte metabolism indicated that the bioactivity of gametocytes appeared to be low already in stage 3 with respect to oxidation stress due to the digestion of Hb.^27^ Quantification of such Hb consumption dynamics may be possible with a time-traced optical measurement of the transition from Hb abundance to Hz abundance, an important step toward a more complete understanding of gametocyte biology.^22^^,^^28^

The hyperspectral imaging technology with an improved data acquisition rate and reduced cost in the future may also offer benefits to resource-limited rural communities for the rapid and accurate detection of malaria as well as in the efficacy testing of antimalarial drugs. The high cost of hyperspectral imaging systems used to be a challenge for fieldable applications in resource-limited communities. However, recent technological advancement in the development of thin film or on chip-based spectral analyzer components has enabled cost-effective and rapid snapshot hyperspectral imaging with comparable or even better capabilities such as spectral bandwidth, resolution, and data acquisition speed. The new snapshot techniques’ acquisition speed is three to four orders of magnitude higher than current technologies. With increasing industrial involvement to integrate the on-chip spectrum-resolving component with a low-cost CMOS imaging sensor, the cost is expected to continue to drop to an affordable level. With the new low-cost high-speed snapshot imaging system, high-throughput data acquisition would be possible.^29^ According to a clinical study in an endemic area,^30^ blood samples from 82.9% of the early-stage febrile patients showed parasite counts of >0.08%, which corresponds to 80,000 parasitized cells in 100 million, which can be imaged in less than 2 min, sufficient for the early detection. Similarly, high-speed imaging for the drug study at the GMP level, if necessary, would be possible. Another cost-effective option is a ratiometric discrete-wavelength spectral image analysis validated by well-calibrated broadband hyperspectral analysis. A discussion on this approach is out of the scope of this study.

Common molecular detection methods including polymerase chain reaction (PCR) and rapid diagnostic test (RDT) kits have been applied to malaria detection in the field. As resource-limited communities may not be able to afford or access PCR technologies, RDT kits are a convenient method but are known to have the potential to miss the detection of mutant parasites with deletions of target genes (e.g., HRP2 and HRP3)^31^^,^^32^ and require periodic quality checks and strict maintenance, creating challenges for field applications in rural areas.^32^ A simple visual or microscopy-based assessment of Giemsa-stained blood smear samples has been used as the gold standard to confirm the presence of parasites in the blood,^33^ offering an affordable solution for rural areas where advanced diagnosis instruments are not available. However, this technique is based on the white-light imaging of whole blood, and the sensitivity is strongly influenced by the imaging conditions, sample preparation and procedures, and numerous other factors. Although characteristic infection stages may be observed under a microscope, there are limitations to discerning them with the human eye, and detailed observations, such as the changes in Hb levels within cells and variations in cellular morphology, can be challenging. In essence, the advantage of our approach over the conventional Giemsa-staining and bright-field microscope approaches is twofold: a spectroscopic imaging capability at a single-cell resolution and further intracellular molecular mapping. A disadvantage is lower data acquisition speed. However, this challenge may be easily overcome by new snapshot hyperspectral imaging techniques discussed above. In this regard, hyperspectral microscopy deployed in the field can provide data cubes that can be analyzed by user-friendly software or online.

## Supplementary Material



## Data Availability

Data and code used in this paper can be made available upon reasonable request to the corresponding author. The materials used in this paper can be made available through a material transfer agreement.
