# Supplementary material for: Hyperspectral analysis to assess gametocytogenesis stage progression in malaria-infected human erythrocytes
Source: J Biomed Opt. 2025 Jan 24;30(2):023516. doi: 10.1117/1.JBO.30.2.023516 (PMC11757776; doi:10.1117/1.JBO.30.2.023516)
Supplement: Supplementary file 1 [file JBO_030_023516_SD001.docx]

**Supplementary Material**

**Hyperspectral analysis to assess gametocytogenesis stage progression in malaria-infected human erythrocytes**

**Ik Hwan Kwon^a,§^, Ji Youn Lee^b,c,§^, Fuyuki Tokumasu^d,e,f^, Sang-Won Lee^a,g^, Jeeseong Hwang^h,^***

**^a^** Nanobio Measurement Group, Division of Biomedical Metrology, Korea Research Institute of Standards and Science, Daejeon 34113, Korea

**^b^** Biometrology Group, Division of Biomedical Metrology, Korea Research Institute of Standards and Science, Daejeon 34113, Korea

^c^ Graduate School of Analytical Science and Technology, Chungnam National University, Daejeon 34134, Republic of Korea

^d^ Department of Laboratory Sciences, Graduate School of Health Sciences, Gunma University, Showa-machi, Maebashi, Gunma 371-8514, Japan

^e^ Department of Cellular Architecture Studies, Division of Shionogi Global Infectious Diseases Division, Institute of Tropical Medicine (NEKKEN), Nagasaki 852-8523, Japan

^f^ School of Tropical Medicine and Global Health, Nagasaki University, Nagasaki 852-8523, Japan

^g^ Department of Applied Measurement Science, University of Science and Technology, Daejeon 34113, Korea

^h^ Applied Physics Division, National Institute of Standards and Technology, Boulder, Colorado 80305, USA

^§^These authors contributed equally.

^*^Corresponding author, E-mail: jeeseong.hwang@nist.gov


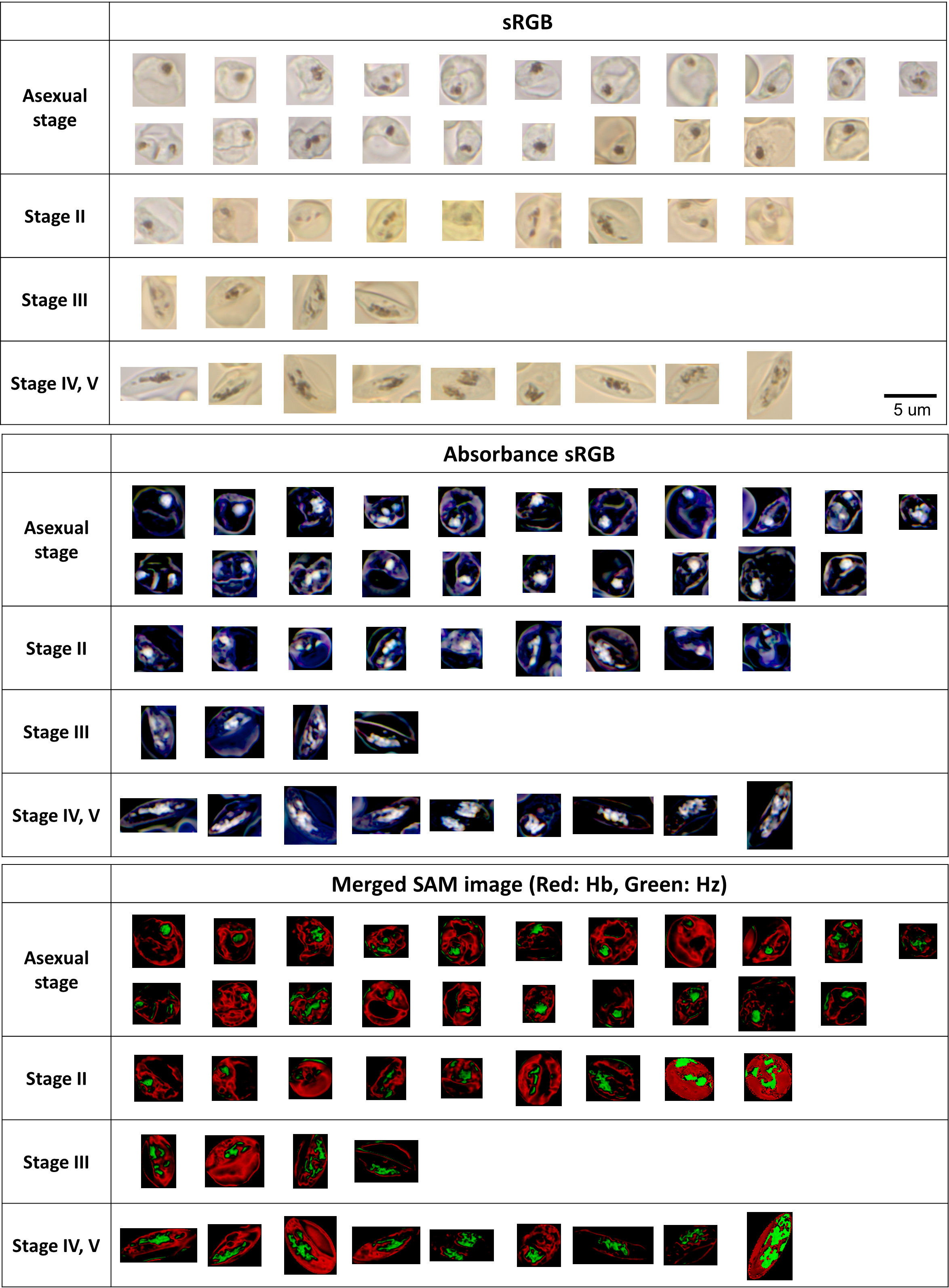


**Figure S1.** sRGB images from the hyperspectral datasets of cells at different stages, absorbance sRGB images, and SAM images merged with segmented maps of Hb and Hz (Hb in red and Hz in green). The same scale bar shown is applied to all images.


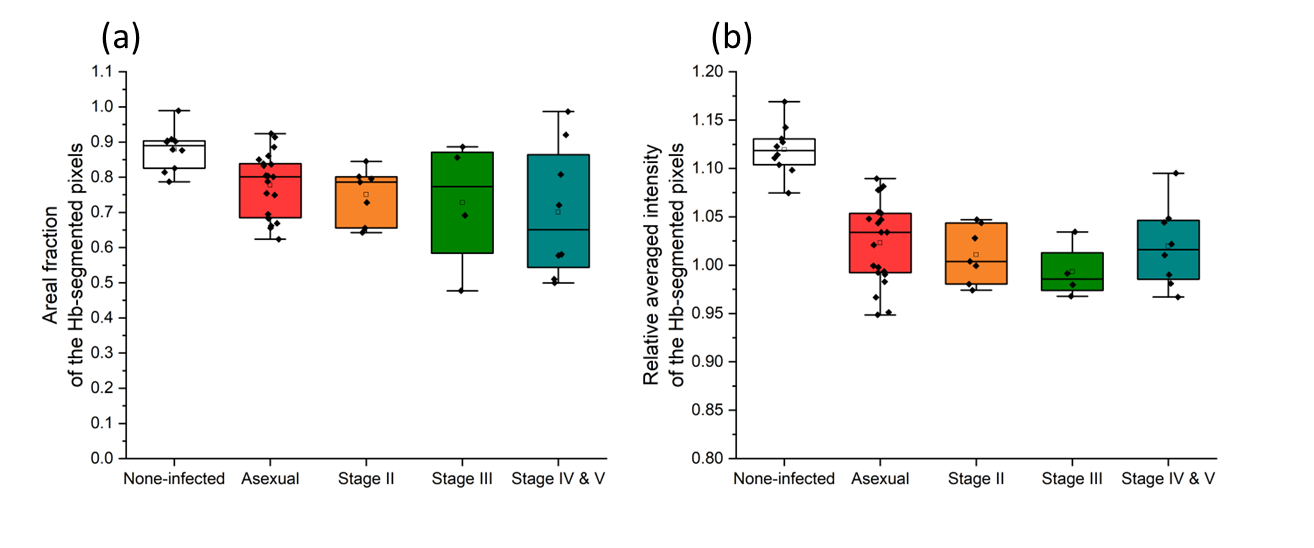


**Figure S2.** Areal fraction vs stage and relative averaged intensity of the Hb-segmented pixels vs stage. (a) A box plot exhibiting the mean value of the Hb-segmented area decreases from the asexual to the gametocyte stage and to the later gametocytogenesis stages. (b) A Box plot exhibiting the mean value of the pixel intensity also decreases from the asexual to the gametocyte stage and to the later gametocytogenesis stages except at the stages IV and V, qualitatively suggesting a trend of increasing Hb consumption as the gametogenesis progresses.
